# Saireito (TJ-114), a Japanese Traditional Herbal Medicine, Reduces 5-Fluorouracil-Induced Intestinal Mucositis in Mice by Inhibiting Cytokine-Mediated Apoptosis in Intestinal Crypt Cells

**DOI:** 10.1371/journal.pone.0116213

**Published:** 2015-01-07

**Authors:** Shinichi Kato, Shusaku Hayashi, Yumeno Kitahara, Koyo Nagasawa, Hitomi Aono, Junichiro Shibata, Daichi Utsumi, Kikuko Amagase, Makoto Kadowaki

**Affiliations:** 1 Division of Pathological Sciences, Department of Pharmacology and Experimental Therapeutics, Kyoto Pharmaceutical University, Misasagi, Yamashina, Kyoto 607–8414, Japan; 2 Division of Gastrointestinal Pathophysiology, Department of Bioscience, Institute of Natural Medicine, University of Toyama, Sugitani, Toyama 930–0194, Japan; Charité, Campus Benjamin Franklin, GERMANY

## Abstract

Clinical chemotherapy frequently causes intestinal mucositis as a side effect, which is accompanied by severe diarrhea. We recently showed that the cytokine-mediated apoptotic pathway might be important for the development of intestinal mucositis induced by 5-fluorouracil (5-FU). Saireito, the traditional Japanese herbal (Kampo) medicine, is widely used to treat diarrhea and various inflammatory diseases in Japan. In the present study, we investigated the effect of saireito on 5-FU-induced intestinal mucositis in mice, especially in relation to apoptosis in the intestinal crypt. Male C57BL/6 mice were given 5-FU (50 mg/kg), i.p. once daily for 6 days. Intestinal mucositis was evaluated histochemically. Saireito (100–1000 mg/kg) was administered p.o. twice daily for 6 days. Repeated 5-FU treatment caused severe intestinal mucositis including morphological damage, which was accompanied by body weight loss and diarrhea. Daily administration of saireito reduced the severity of intestinal mucositis in a dose-dependent manner. Body weight loss and diarrhea during 5-FU treatment were also significantly attenuated by saireito administration. The number of apoptotic and caspase-3-activated cells in the intestinal crypt was increased, and was accompanied by up-regulated tumor necrosis factor (TNF)-α and interleukin (IL)-1β mRNA within 24 h of the first 5-FU injection. However, all of these measures were significantly lower after saireito administration. These results suggest that saireito attenuates 5-FU-induced intestinal mucositis. This action may come from the reduction of apoptosis in the intestinal crypt via suppression of the up-regulation of inflammatory cytokines. Therefore, saireito may be clinically useful for the prevention of intestinal mucositis during cancer chemotherapy.

## Introduction

Intestinal mucositis is a common side effect of clinical chemotherapy for patients with cancer [1, 2], and includes symptoms such as severe diarrhea and dehydration. These symptoms can lead to worsened systemic conditions [2–4]. Although bone-marrow toxicity during chemotherapy can be managed by supportive care, blood transfusion, and colony-stimulating factor treatment, there is currently no beneficial treatment or preventive measure for intestinal mucositis [5]. Therefore, intestinal mucositis is a limiting factor for effective cancer chemotherapy.

Although the antimetabolite anticancer agent, 5-fluorouracil (5-FU), is widely used to treat several types of malignant tumors, it frequently causes intestinal mucositis. The pathogenic mechanisms of 5-FU-induced intestinal mucositis are still unclear, but several pathogenic elements are considered to be involved, including direct toxicity, oxidative stress, apoptosis, hypoproliferation, and abnormal inflammation [6–9]. Particularly, apoptosis is a critical event in the occurrence of 5-FU-induced intestinal mucositis [1, 5, 10, 11], since many apoptotic cells are observed in intestinal crypts before serious mucosal destruction in mice and humans [1, 12, 13]. We recently demonstrated that apoptosis is detected in intestinal crypts 24 h after the first administration of 5-FU in mice [13]. The up-regulation of inflammatory cytokines such as tumor necrosis factor (TNF)-α and interleukin (IL)-1β was similarly observed within 24 h of 5-FU treatment. We also found that 5-FU-induced apoptosis in the crypt was dependent on the up-regulation of these cytokines, since 5-FU-induced apoptosis was potently attenuated by inhibiting cytokine expression [14]. These findings suggest that cytokine-mediated apoptosis may play a critical role in the pathogenesis of 5-FU-induced intestinal mucositis. Early apoptosis may induce intestinal crypt hypoplasia, leading to increased permeability via the destruction of the mucosal barrier. This destruction may lead to increased susceptibility to infection against intestinal bacteria.

Saireito, a traditional Japanese herbal medicine, is a combined formulation of two herbal medicines (shosaikoto and goreisan) and is often used to treat inflammatory diseases such rheumatoid arthritis, systemic lupus erythematodes, and nephrotic syndrome [15, 16]. Saireito has been reported to ameliorate the symptoms associated with ulcerative colitis like diarrhea and hematochezia, improve endoscopic observation, and reduce the dosage of corticosteroids necessary in combination therapy [17]. It is therefore possible that saireito may be effective for intestinal mucositis induced by chemotherapeutic agents. Daikenchuto, a frequently prescribed traditional Japanese herbal medicine, is used for treatment of various gastrointestinal motility disorders [18, 19]. In addition, this herbal medicine has been shown to have an anti-inflammatory effect in experimental models of colitis and intestinal mucositis [20–22].

In the present study, we investigated the effect of saireito on 5-FU-induced intestinal mucositis in mice in comparison with daikenchuto, especially focusing on its relationship with cytokine-mediated apoptosis in the intestinal crypt.

## Materials and Methods

### Animals and ethics statement

This study was carried out in strict accordance with the recommendations in the Guide for Care and Use of Laboratory Animals of the National Institutes of Health. The protocols were approved by the committee on the Ethics of Animal Research of Kyoto Pharmaceutical University (Permit Number: 13–010). Eight to nine-week-old male C57BL/6 mice weighing 20–24 g were purchased from SLC incorporation (Shizuoka, Japan). All mice were maintained in plastic cages with free access to food and water, and housed at 22 ± 1°C with a 12-h light/dark cycle.

### Drugs

**Figure 1:**
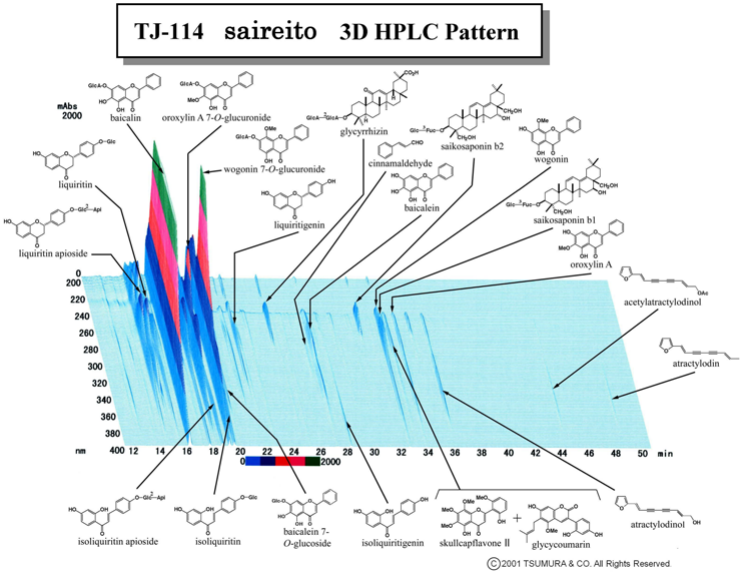
Three-dimensional high-performance liquid chromatograph pattern of saireito (TJ-114).

5-FU was purchased from Sigma–Aldrich (St. Louis, MO, USA). Methylcellulose (CMC) was purchased from Nacali Tesque (Kyoto, Japan). Saireito (TJ-114) and daikenchuto (TU-100) are the prescription drugs covered under the National Health Insurance Plan in Japan and were provided by Tsumura & Co. (Tokyo, Japan). According to the manufacturer’s information, saireito is a water extract of 6.0 g of herbal powder. Saireito extract is prepared from a decoction of the following 12 medicinal herbs: *Bupleuri radix*, 7.0 g; *Pinelliae tuber*, 5.0 g; *Alismatis rhizome*, 5.0 g; *Scutellariae radix*, 3.0 g; *Ginseng radix*, 3.0 g; *Zizyphi fructus*, 3.0 g; *Poria*, 3.0 g; *Polyporus*, 3.0 g; *Atractylodis lanceae rhizome*, 3.0 g; *Cinnamomi cortex*, 2.0 g; *Glycyrrhizae radix*, 2.0 g; and *Zingiberis rhizome*, 1.0 g. Daikenchuto is a water extract of 1.5 g of herbal powder with the addition of maltose syrup powder (88.9%). Daikenchuto extract is prepared from a decoction of the following three medicinal herbs: *Zanthoxylum Fruit*, 2.0 g; *Ginseng*, 3.0 g; and processed *Ginger*, 5.0 g. The three-dimensional high-performance liquid chromatograph (3D-HPLC) profile of saireito provided by Tsumura & Co. is shown in Fig. 1. 5-FU was dissolved in physiological saline at a concentration of 5 mg/mL. Saireito (100, 300, and 1000 mg/kg) and daikenchuto (2700 mg/kg) were suspended in 0.5% carboxymethylcellulose (CMC) solution at concentrations of 10, 30, 100, and 270 mg/ml. All drugs were prepared immediately before use and administered i.p. or p.o. at a volume of 0.1 mL/10 g body weight [23].

### Induction of intestinal mucositis

Intestinal mucositis was induced by 6 days of 5-FU i.p. injection (50 mg/kg). The animals were divided into six experimental groups: normal (n = 8), control (n = 6), 100 mg/kg of saireito (n = 8), 300 mg/kg of saireito (n = 6), 1000 mg/kg of saireito (n = 8), and 2700 mg/kg of daikenchuto (n = 6). Saireito and daikenchuto were administered p.o. twice, 30 min before and 8 h after 5-FU injection for 6 days (day 0 to 5). The normal group received physiological saline (the vehicle of 5-FU) and CMC solution (the vehicle of saireito/daikenchuto). The control group was given 5-FU and CMC solution (the vehicle of saireito/daikenchuto). The dose of daikenchuto was chosen based on previous study [24]. Disease activity was assayed daily by measuring body weight and scoring the stool consistency as we previously described [14].

### Assessment of intestinal mucositis

On day 6, animals were sacrificed by CO_2_ gas inhalation, the jejunum was removed, and immersed in 10% neutralized formalin overnight. The tissues were embedded in paraffin, cut into 4-µm-thick sections, and stained with hematoxylin and eosin (H&E). The villus height and crypt damage were measured under light microscope at a magnification of 100× and 400×, respectively (BX-51; Olympus, Tokyo, Japan), as previously described [14].

### Determining apoptosis, caspase-3 activation, and cell proliferation

On day 1, the jejunum tissues were fixed with 10% neutralized formalin, embedded in paraffin, and cut into 4-µm-thick sections. Apoptosis was detected by TUNEL assay using an *in situ* apoptosis detection kit (Takara, Shiga, Japan) after treatment with proteinase K (Takara). Caspase-3 activation and cell proliferation were determined immunohistochemically using rabbit anti-cleaved caspase-3 (Cell Signaling Technology, Danvers, MI, USA) and rabbit anti-Ki-67 (Novus Biologicals, Littleton, CO, USA) antibodies, respectively, after treatment with HistoVT One (Nacalai Tesque, Kyoto, Japan) and 3% hydrogen peroxide (to block endogenous peroxidases). Immune positive cells were visualized with the Vectastain Elite ABC rabbit IgG kit (Vector Laboratories, Burlingame, CA, USA). Sections were counter-stained with hematoxylin. The number of cells positive for TUNEL assays, cleaved caspase-3, and Ki-67 immunostainings were counted under a light microscope at a magnification of 400×, as previously described [14].

### Determination of TNF-α and IL-1β mRNA expression by quantitative RT-PCR

On day 1, jejunum tissue was washed with cold phosphate buffered saline (PBS), and stored in RNA later (Ambion, Austin, TX, USA) at 4°C before use. Total RNA was extracted using Separose RNA-I Supper G (Nacalai Tesque) and reverse transcription (RT) was performed using PrimeScript Reverse Transcriptase (Takara). Quantitative polymerase chain reaction (PCR) was carried out using ABI 7500 (Applied Biosystems, Foster City, CA, USA) with SYBR Premix ExTaq (Takara). Specific primer sets of β-actin (MA050368), TNF-α (MA097070), and IL-1β (MA025939) were obtained from the Perfect Real-Time Supporting System (Takara). Expression levels of TNF-α and IL-1β mRNA were calculated using the comparative CT (ΔΔC_T_) method (normalized to the mean value of the normal group).

### Anti-tumor effect of 5-FU in tumor-implanted mice

To examine the influence of saireito on the anti-tumor effect of 5-FU, Colon 38 tumor-implanted mice were prepared as previously described [25]. The animals were divided into three experimental groups: normal (n = 7), control (n = 6), and 1000 mg/kg of saireito (n = 8). 5-FU (20 mg/kg) was administered i.p. once daily for 6 days (days 7–12), starting from the 7^th^ day after tumor implantation. Saireito (1000 mg/kg) was administered p.o. twice daily for 14 days (days 7–21), starting from the 7^th^ day after the implantation. The normal group received physiological saline (the vehicle for 5-FU) and CMC solution (the vehicle for saireito). The control group was given 5-FU and CMC solution (the vehicle for saireito). The solid tumor volume and body weight were measured every 2 or 3 days for 14 days (days 7–21), starting 7 days after the implantation.

### Statistical analysis

Data are presented as the mean ± SEM. Statistical analyses were analyzed with GraphPad Prism 6.0b (GraphPad Software, La Jolla, CA, USA) using a parametric one-way ANOVA followed by Dunnett’s multiple comparison test and non-parametric Kruskal-Wallis one-way ANOVA followed by Dunn’s multiple comparison test, with P<0.05 regarded as statistically significant.

## Results

### Effect of saireito on body weight loss and diarrhea during 5-FU treatment

**Figure 2:**
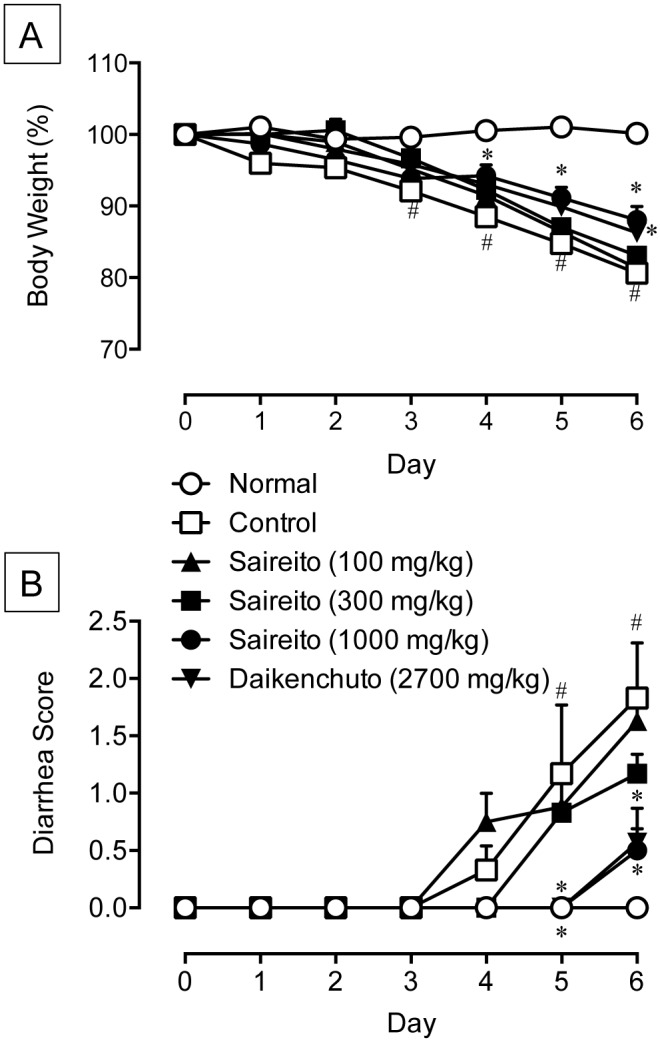
Effect of saireito and daikenchuto on changes in body weight and diarrhea during 5-fluorouracil (5-FU) treatment. 5-FU (50 mg/kg) was injected i.p. once daily while saireito (100–1000 mg/kg) and daikenchuto (2700 mg/kg) were administered p.o. twice daily for 6 days (days 0–5). Body weight is shown as a percentage of initial body weight (A), whereas the severity of diarrhea is scored using the four-grade scale (0 to 3) (B). Data are presented as the mean ± SEM of 6–8 mice. *P < 0.05 versus control (vehicle alone), ^#^ *P < 0.05* versus normal (5-FU-untreated).

Repeated administration of 5-FU (50 mg/kg) to animals caused body weight loss and diarrhea (Fig. 2A and B). Significant body weight loss was observed on day 3, and the mean body weight was reduced to 80.6 ± 1.7% of initial body weight on day 6. Prominent diarrhea was observed on day 4, and the mean diarrhea score reached 1.8 ± 0.53 on day 6. Twice-daily administration of saireito (100–1000 mg/kg) dose-dependently reduced body weight loss and diarrhea during 5-FU treatment. A significant effect on body weight loss and diarrhea was observed at a dose of 1000 mg/kg. Daikenchuto (2700 mg/kg), when given twice daily, also significantly reduced 5-FU-induced body weight loss and diarrhea. This effect was similar to that of saireito at 1000 mg/kg.

### Effect of saireito on 5-FU-induced intestinal mucositis

**Figure 3:**
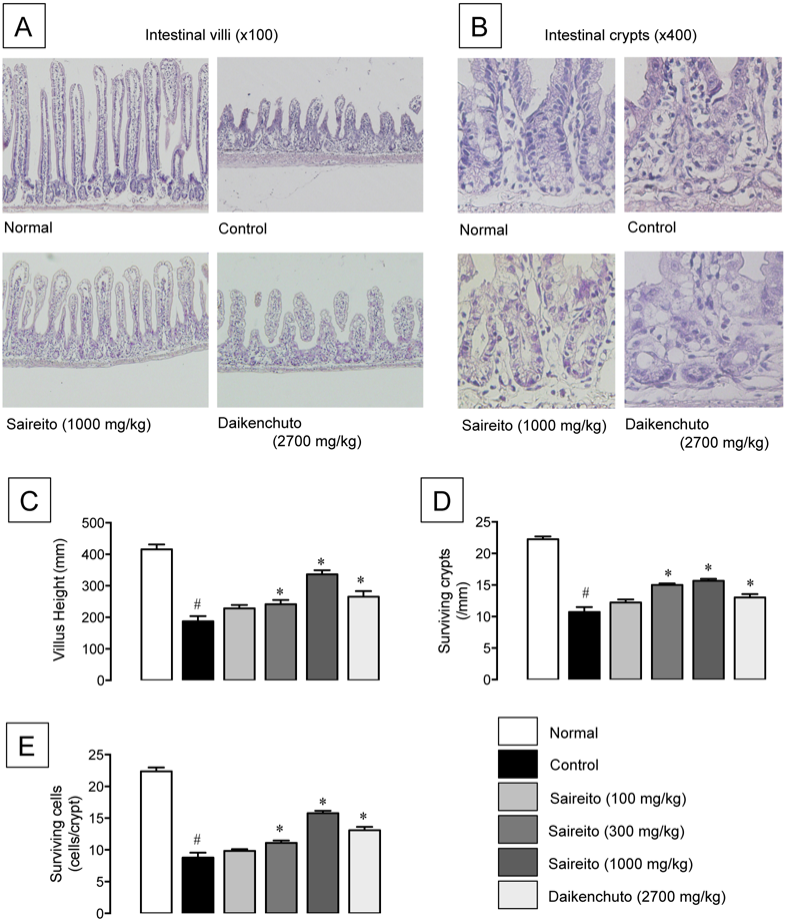
Effect of saireito on shortening of villus height and crypt destruction induced by 5-fluorouracil (5-FU) in mouse small intestines. 5-FU (50 mg/kg) was injected i.p. once daily while saireito (100–1000 mg/kg) and daikenchuto (2700 mg/kg) were administered p.o. twice daily for 6 days (days 0–5). The jejunum was excised on day 6, sectioned, and stained with H&E. Histological observations for intestinal villi (A, 100×) and crypts (B, 400×) were performed. The height of villi (C), the number of surviving crypts per millimeter (D), and surviving cells per crypt (E) were measured. Data are presented as the mean ± SEM of 6–8 mice. *P < 0.05, versus control (vehicle alone); ^#^ *P < 0.05*, versus normal (5-FU-untreated).

Repeated administration of 5-FU (50 mg/kg) caused severe intestinal mucositis, characterized by shortened villus height (Fig. 3A) and crypt destruction (Fig. 3B). The villus height shortened to less than half that of the normal value on day 6 (Fig. 3C). The destruction of intestinal crypts was evaluated by a decrease in the number of surviving crypts and crypt cells. The number of surviving crypts and crypt cells decreased to 48.2 and 39.3% that of normal values, respectively, on day 6 (Fig. 3D and E). Twice-daily administration of saireito (100–1000 mg/kg) dose-dependently mitigated the morphological changes induced by 5-FU, and significant effects were observed at more than 300 mg/kg. In contrast, when daikenchuto (2700 mg/kg) was administered twice daily, there was significantly less intestinal mucositis caused by 5-FU, but the effect was less than that of saireito.

### Effect of saireito on 5-FU-induced apoptosis in the intestinal crypt

**Figure 4:**
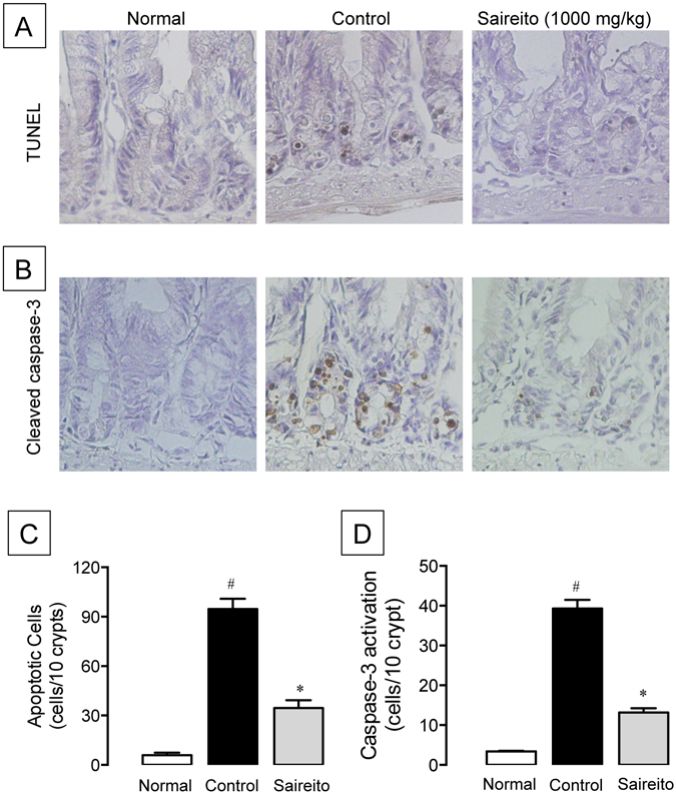
Effect of saireito on apoptosis and caspase-3 activation in the intestinal crypt induced by 5-fluorouracil (5-FU). 5-FU (50 mg/kg) was injected i.p. while saireito (1000 mg/kg) was administered p.o. twice, 30 min before and 8 h after 5-FU injection. The jejunum was excised 24 h after 5-FU injection, sectioned, and TUNEL assay (A, 400×) and cleaved-caspases-3 immunostaining (B, 400×) were performed. The number of apoptotic (C) and caspase-3-activated cells (D) were counted. Data are presented as the mean ± SEM of 6 mice. *P < 0.05, versus control (vehicle alone); ^#^ *P < 0.05*, versus normal (5-FU-untreated).

We previously reported that apoptosis in the intestinal crypt is most prominently observed 24 h (day 1) after the first administration of 5-FU [13]. In the present study, we also found that administration of 5-FU caused a marked increase in TUNEL-positive apoptotic cells on day 1 (Fig. 4A). The number of apoptotic cells in control animals (5-FU-treated) was about 16-fold that of normal animals (5-FU-untreated) (Fig. 4C). The administration of saireito (1000 mg/kg) twice significantly reduced 5-FU-increased apoptotic cells, with an inhibition rate of 67.8%.

To further investigate the apoptotic response induced by 5-FU, the activation of caspase-3 was assessed immunohistochemically on day 1. Administration of 5-FU caused a marked activation of caspase-3 in the intestinal crypt on day 1 (Fig. 4B). The number of caspase-3-activated cells in control animals was more than 10-fold that of normal animals (Fig. 4D). Administration of saireito (1000 mg/kg) significantly suppressed the activation of caspase-3, with an inhibition rate of 73.0%.

### Effect of saireito on up-regulation TNF-α and IL-1β mRNA induced by 5-FU in small intestine

**Figure 5:**
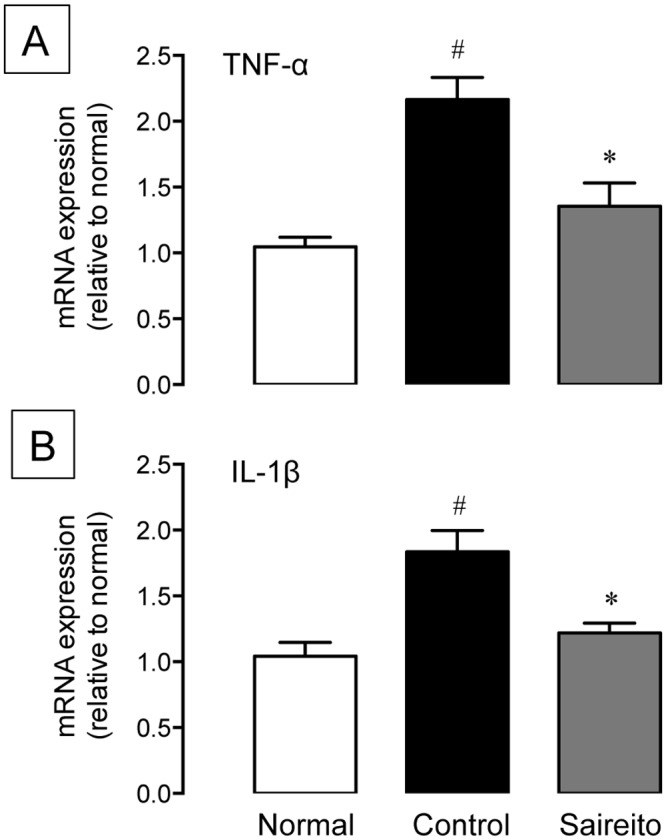
Effect of saireito on increased TNF-α and IL-1β mRNA expression induced by 5-fluorouracil (5-FU) in mouse small intestines. 5-FU (50 mg/kg) was injected i.p. while saireito (1000 mg/kg) was administered p.o. twice, 30 min before and 8 h after 5-FU injection. The jejunum was excised 24 h after 5-FU injection and the expression of TNF-α (A) and IL-1β (B) mRNA was determined by quantitative RT-PCR. Data are presented as the mean ± SEM of 6 mice. *P < 0.05, versus control (vehicle alone); ^#^ *P < 0.05*, versus normal (5-FU-untreated).

We previously demonstrated that the apoptotic pathways mediated by cytokines such as TNF-α and IL-1β are involved in 5-FU-induced intestinal crypt cell apoptosis [13, 14]. In the present study, we also observed an approximately 2-fold increase in TNF-α and IL-1β mRNA expressions on day 1 (Fig. 5A and B). The administration of saireito (1000 mg/kg) significantly attenuated the up-regulation of both TNF-α and IL-1β mRNA induced by 5-FU. The inhibition rate was 72.5 and 77.6% for TNF-α and IL-1β, respectively.

### Effect of saireito on 5-FU-induced anti-proliferative action in the intestinal crypt

**Figure 6:**
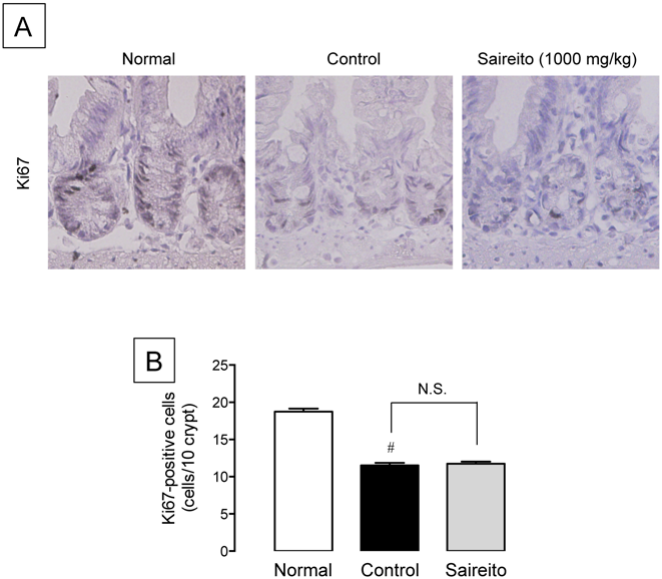
Effect of saireito on the suppression of cell proliferation induced by 5-fluorouracil (5-FU) in mouse small intestines. 5-FU (50 mg/kg) was injected i.p. while saireito (1000 mg/kg) was administered p.o. twice, 30 min before and 8 h after 5-FU injection. The jejunum was excised, sectioned, and Ki67 immunostaining was performed (A). The number of proliferative cells was counted (B). Data are presented as the mean ± SEM of 6 mice. ^#^ *P < 0.05*, versus normal (5-FU-untreated).

To confirm the influence of saireito on the anti-proliferative action of 5-FU in intestinal crypt cells, the cell proliferative activity was determined immunohistochemically. A large number of Ki67-positive proliferative cells were detected in the intestinal crypt of normal animals. The administration of 5-FU significantly reduced the number of Ki67-potitive cells to 61.3% that of normal animals (Fig. 6A and B). The administration of saireito (1000 mg/kg) did not affect the anti-proliferative action of 5-FU.

### Effect of saireito on anti-tumor action of 5-FU in Colon 38 tumor-implanted mice

**Figure 7:**
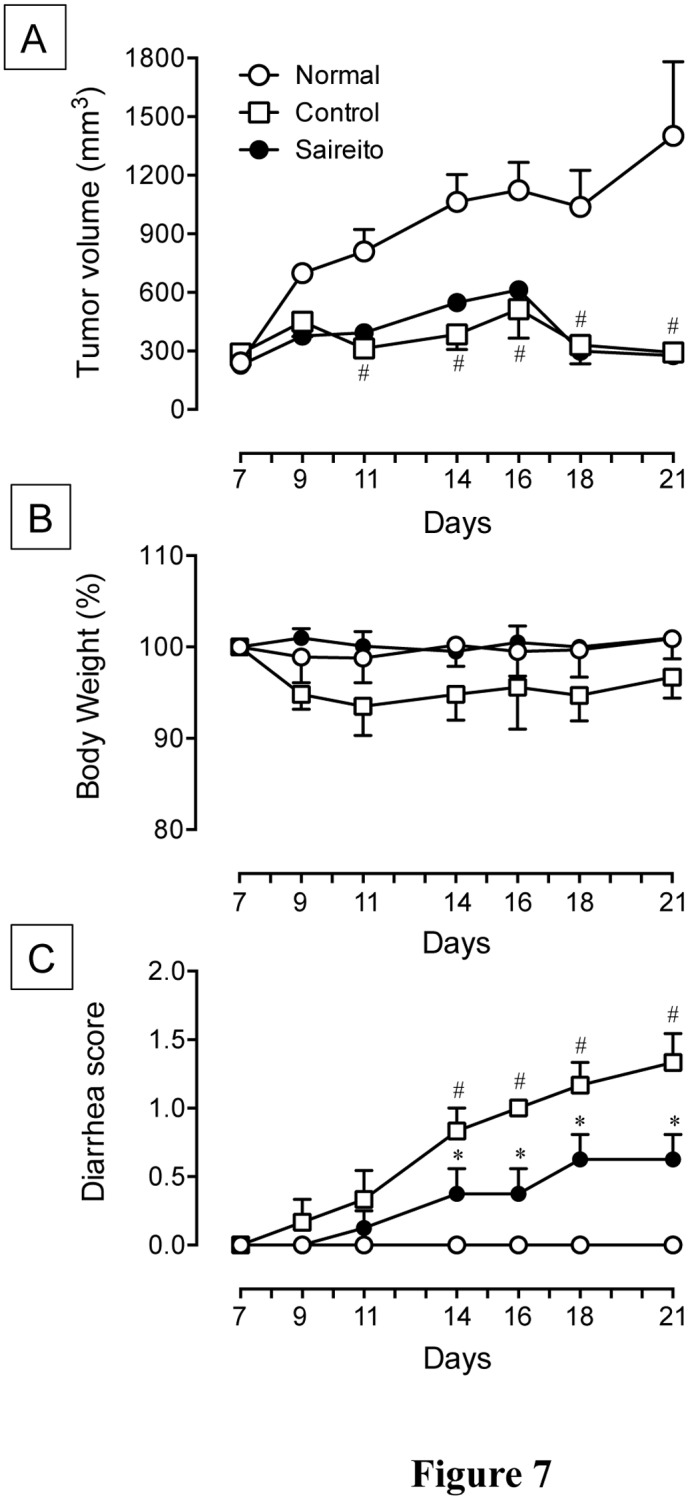
Effect of saireito on the anti-tumor action, body weight loss, and diarrhea induced during 5-fluorouracil (5-FU) treatment in Colon 38 tumor-implanted mice. 5-FU (20 mg/kg) was injected i.p. once daily for 6 days (days 7–12), while saireito (1000 mg/kg) was administered p.o. twice daily for 14 days (days 7–21), starting 7 day after tumor implantation. The volume (mm^3^) of solid tumor (A), body weight (B), and diarrhea score (C) were determined every 2 or 3 days, starting 7 days after the implantation. Data are presented as the mean ± SEM of 6–8 mice. *P < 0.05, versus control (vehicle alone); ^#^ *P < 0.05*, versus normal (5-FU-untreated).

To confirm the influence of saireito on the anti-tumor action of 5-FU, we administered saireito to Colon 38 tumor-implanted mice. Subcutaneous implanted Colon 38 tumor fragments developed single and solid tumors, and the average volume of the solid tumor increased to 1400 mm^3^ by day 21 following tumor implantation (Fig. 7A). Repeated administration of 5-FU (20 mg/kg) for 14 days (days 7–14) potently reduced tumor growth during the experimental period. Twice-daily administration of saireito (1000 mg/kg) did not affect the anti-tumor action of 5-FU. However, 5-FU at a low dose (20 mg/kg) caused body weight loss and diarrhea in Colon 38-implanted mice (Fig. 7B and C). The administration of saireito significantly decreased the severity of diarrhea and trended towards a prevention of body weight loss induced by 5-FU.

## Discussion

5-FU is one of the most commonly used chemotherapeutic agents for the treatment of malignant tumors. However, it frequently causes intestinal mucositis accompanied with severe diarrhea [2, 3]. These serious side effects impair quality of life in patients and can lead to discontinuation of cancer chemotherapy. Therefore, immediate and effective countermeasures against 5-FU-induced intestinal mucositis are necessary. In the present study, we found that the Japanese traditional herbal medicine, saireito, contributes to a reduction in 5-FU-induced intestinal mucositis. Since saireito is currently used in Japan for the treatment of various inflammatory diseases such as rheumatoid arthritis, systemic lupus erythematodes, and nephrotic syndrome [15, 16], this finding is clinically interesting and relevant.

Several studies have demonstrated that the administration of 5-FU to experimental animals produced diarrhea and body weight loss accompanied by morphological damage to the small intestine [2–4]. The present findings are consistent with those of our previous study [13], indicating that repeated administration of 5-FU (50 mg/kg) to mice causes severe intestinal mucositis. The mucositis is morphologically characterized by the shortening of villus height and destruction of crypts in the small intestine [13, 14]. Twice-daily administration of saireito dose-dependently reduced the severity of 5-FU-induced morphological intestinal damage and clinical symptoms such as diarrhea and body weight loss. 5-FU-induced diarrhea is closely associated with intestinal mucositis severity, and the deterioration of systemic conditions followed by diarrhea and dehydration results in body weight loss [4]. Therefore saireito could be effective against 5-FU-induced intestinal mucositis and its related clinical symptoms.

Daikenchuto has been shown to improve gastrointestinal motility, postoperative adhesion, and ileus in experimental animals [18, 24, 26]. Several studies also demonstrated that daikenchuto has an anti-inflammatory effect in experimental models of colitis including trinitrobenzenesulfonic acid-induced, helper type-1 T cell (Th1)- [20] and oxazolone-induced helper type-2 T cell (Th2)-dominant colitis [21], naive CD4^+^ T cell transfer [27], and intestinal mucositis induced by chemotherapeutic agent irinotecan [22]. In the present study, we observed that daikenchuto exerts a similar effect on body weight loss and diarrhea induced by 5-FU. However, the preventive effect of daikenchuto on morphological damage induced by 5-FU was less than that of saireito. Therefore, saireito is more effective and possibly more useful for the preventing and treating 5-FU-induced intestinal mucositis.

Although the pathogenesis of 5-FU-induced intestinal mucositis is not fully understood, several pathogenic elements in addition to direct cytotoxicity are considered to be involved, including apoptosis, hypoproliferation, and abnormal inflammation [7–9]. Particularly, apoptosis is a critical event in intestinal mucositis induced by 5-FU [1, 5, 10, 12, 13]. We previously reported that apoptosis was confined to intestinal crypts and detected within 24 h of the first 5-FU challenge [13]. Moreover, 5-FU-induced apoptosis was detected preceding the onset of morphological damage. These findings strongly suggest that apoptosis in the intestinal crypt is important in the initiation of 5-FU-induced intestinal mucositis. In the present study, we observed that the number of TUNEL-positive apoptotic cells in the intestinal crypt on day 1 was significantly lower after administration of saireito. Saireito similarly attenuated the activation of caspase-3 in response to 5-FU in the intestinal crypt. Caspase-3, the main downstream effector and a key factor in the process of apoptosis [28, 29], is considered to be involved in 5-FU-induced apoptosis in the intestinal crypt [30–32]. These findings suggest that saireito significantly reduces 5-FU-induced intestinal mucositis via inhibition of the apoptotic response in the intestinal crypt.

Apoptosis is triggered by proteolytic enzymes such as caspases [33]. Activation of caspases in response to cancer chemotherapy can be initiated via two signaling pathways, intrinsic and extrinsic [34]. The intrinsic pathway is generally activated by DNA damage and when pro-apoptotic proteins such as *Bax* are released from mitochondria. In contrast, the extrinsic pathway is initiated outside of cells via activation of death receptor signaling such as from TNF-α. Several studies demonstrated that apoptosis induced by 5-FU at high doses (130–200 mg/kg) is accompanied by increased pro-apoptotic *Bax* expression and decreased anti-apoptotic *Bcl-2* expression through the intrinsic apoptotic pathway [12, 30]. We recently found, however, that repeated administration of 5-FU at a low dose (50 mg/kg) failed to affect the expression of these pro- and anti-apoptotic proteins [14]. We further showed that caspase-8 was potently activated in intestinal crypt cells 24 h after the first administration of 5-FU. Caspase-8 is an initiator that promotes caspase-3 activation, and the activation of this enzyme is initiated by members of the TNF family via the extrinsic apoptotic pathway. Therefore, apoptosis induced in the intestinal crypt by low doses of 5-FU is probably mediated by activation of extrinsic cytokine-mediated apoptotic pathways. Indeed, several studies have demonstrated that 5-FU-induced apoptosis was potently prevented by inhibiting cytokine expression [14, 30, 35]. In the present study, administration of 5-FU caused up-regulation of inflammatory cytokines such as TNF-α and IL-1β in the small intestine. Interestingly, saireito significantly attenuated up-regulation of these cytokines. These findings suggest that saireito reduces 5-FU-induced apoptosis by inhibiting the cytokine-mediated apoptotic pathway.

Several studies have demonstrated that saireito has immunomodulatory effects on various autoimmune diseases. In MRL/lpr mice after administration of saireito, Th1-dominant systemic lupus nephritis was ameliorated via increased Th2-cytokines, such as IL-4, and decreased Th1-cytokines, such as interferon (IFN)-γ [16]. Similarly, in Th1-dominant autoimmune uveitis, saireito decreased the production of IFN-γ [36]. In contrast, saireito has been shown to reduce the severity of Th2-dominant oxazolone-induced colitis via suppression of Th2-cytokine production [23]. Therefore, saireito may have immunomodulatory effects via modulation of the Th1/Th2 balance, depending on the type of autoimmune disease. Saireito can also reduce the production of cytokines such as TNF-α, IL-1β, and transforming growth factor beta [36, 37]. Saikosaponins, a well-known active component of saireito, suppressed inflammatory cytokine production in macrophages via inhibition of nuclear factor-kappa B and mitogen-activated protein kinase [38, 39]. Similarly, *Scutellaria radix*, an herbal constituent of saireito, has an anti-inflammatory effect in LPS-activated macrophages [40]. In the 3D-HPLC profile (Fig. 1), we found that saireito includes a large amount of baicalin, wogonin, and their related derivatives, and these components come from *Scutellaria radix*. Further studies are needed to elucidate the detailed mechanisms by which saireito suppresses cytokine production, including the relationship to active components.

We also examined the influence of saireito on the anti-tumor action of 5-FU in mice implanted with Colon 38 tumors, a murine colon adenocarcinoma cell line. Repeated administration of 5-FU totally reduced the growth of solid tumors, and the anti-tumor action of 5-FU was not affected by daily administration of saireito. In fact, twice-daily administration of saireito prevented diarrhea and body weight loss during low dose 5-FU treatment (20 mg/kg) in Colon 38-implanted mice. Moreover, 5-FU decreased cell proliferation, as determined by Ki67 immunostaining in the intestinal crypt, but this response was not affected by saireito. These findings strongly suggest that saireito has no influence on the anti-proliferative and anti-tumor actions of 5-FU. Therefore, it is likely that saireito may be able to ameliorate intestinal mucositis without negative influences on the anti-tumor action of 5-FU during chemotherapy.

## Conclusions

Saireito can reduce 5-FU-induced intestinal mucositis through reduction of apoptosis in the intestinal crypt via suppression of the up-regulation of inflammatory cytokines. Therefore, saireito may be clinically useful for preventing and treating intestinal mucositis during cancer chemotherapy.
